# Gender, Living Conditions, and Family Support Associated with Depression Status among Adolescents in Semarang, Indonesia

**DOI:** 10.2174/0117450179467270260709105016

**Published:** 2026-07-13

**Authors:** Meira Erawati, Anggorowati, Innez Karunia Mustikarani, Apri Nur Wulandari, Fihris Aulia Sa’adah, Alfina Shafira Difany

**Affiliations:** 1 Department of Nursing, Faculty of Medicine, Universitas Diponegoro, Tembalang, Semarang, Central Java, Semarang, Indonesia

**Keywords:** Adolescent, Depression, Determinants, Family support, Gender, Living condition

## Abstract

**Introduction:**

Adolescents around the world encounter profound difficulties with depression. Adolescents exhibit a vulnerability to depression stemming from a variety of influences, encompassing sociodemographic factors and the level of familial support available to them. However, research on the specific association of factors and depression among adolescents remains limited in Semarang. This study aimed to examine the association between sociodemographic factors and family support with depression among adolescents in Semarang, Indonesia.

**Method:**

This research employed a quantitative approach within a cross-sectional design. We employed proportional stratified random sampling to select our sample and gathered data from 421 respondents in Semarang utilizing the sociodemographic questionnaire, the PSS-Fa (Perceived Social Support Family), and PHQ-9 (Patient Health Questionnaire-9). The data analysis encompasses univariate, bivariate, and multivariate analyses. Univariate analysis was conducted using a frequency distribution table, whereas bivariate analysis employed the chi-square and Fisher's exact test. Meanwhile, multivariate analysis was conducted using Multivariate ordinal logistic regression analysis. The significant value was less than 0.05.

**Result:**

The results demonstrated that the predominant age group among adolescents was 16 years (61.8%), with female participants accounting for 60.6%. Moreover, 95.7% of adolescents lived with their parents. Most respondents (57%) were in the 10th grade, and 53.9% were considered to have high socioeconomic status. Additionally, more than half of the respondents (51.8%) reported receiving substantial family support. The correlation test results showed a strong link between depression level and a number of factors, such as gender (*p*=0.000), living conditions (*p*=0.037), and family support (*p*=0.000). Meanwhile, education level (*p* = 0.279) and socioeconomic status (*p* = 0.577) were not correlated with depression among adolescents. Multivariate analysis revealed that gender (AOR = 1.79, 95% CI: 1.15–2.80, *p* = 0.011) and family support (AOR = 0.54, 95% CI: 0.38–0.77, *p* = 0.001) were significantly associated with depression.

**Discussion:**

The findings indicated that several factors associated with the depressive state among adolescents in Semarang include gender, living conditions, and family support.

**Conclusion:**

Health service providers need to consider gender, living conditions, and family support when developing health programs for adolescents. Future research is encouraged to explore additional factors and to employ qualitative approaches to further investigate the psychological conditions of adolescents.

## 1 INTRODUCTION

Adolescence is a developmental stage that bridges childhood and adulthood [1], characterized by significant physical, psychological, and social transformations [2]. The transition from childhood to adolescence necessitates the navigation of numerous transitions. Some adolescents have no trouble getting through this change, while others have serious mental health problems [3]. Adolescents often experience stress, despair, and anxiety, making them particularly vulnerable to mental health problems. Depression is one of the most common mental health disorders in this age group [4] and represents a significant public health concern. It can trigger various maladaptive behaviours that may jeopardize adolescents’ well-being and safety. Moreover, depression is a significant factor contributing to self-harm and is a leading cause of suicide among adolescents [5, 6].

In Indonesia, a considerable proportion of adolescents experience depression at varying levels of severity, ranging from mild to severe [7]. The prevalence is particularly concerning in major urban areas, such as Medan, where it has reached a substantial level [8]. Notably, depression is also prevalent among early adolescents, especially those in junior high school [9]. Exposure to violence, whether physical or psychological, has been identified as an important risk factor contributing to depression in this population [10].

Depression is attributable to a multitude of factors, among which sociodemographic status is significant. Sociodemographic categories include gender, age, educational attainment, living conditions, and socioeconomic status [11]. Living conditions, specifically, constitute a sociodemographic factor that can induce depression in adolescents [12]. People show different personality traits when they adapt to the environments in which they are developing. Adolescents living with both parents are more adept at managing depressive symptoms than those whose parents are separated [13]. Educational attainment also plays a role in depression. Previous research conducted indicated a higher incidence of depression among high school adolescents than those in lower educational levels, such as elementary and junior high schools [14]. Individuals with lower socioeconomic status were more likely to experience mental health problems than those with higher socioeconomic status [15]. Family support is defined as the acceptance shown to a family member by other members of the family [16]. Previous studies have reported a negative correlation between social support and depressive symptoms, indicating that adolescents with strong family support are less likely to experience depression [17].

To provide a stronger conceptual foundation, this study is grounded in the Social Determinants of Health (SDH) Framework, complemented by the Stress-Buffering Model of Social Support and the Ecological Systems Theory. These frameworks collectively explain how individual, social, and environmental factors interact to influence adolescent mental health, particularly depression [18–21]. Within the Social Determinants of Health (SDH) Framework, health outcomes are shaped by a combination of structural and intermediary determinants [22]. In this study, key demographic variables are conceptualized as follows: Age and gender are positioned as structural determinants, reflecting inherent and socially constructed characteristics that influence adolescents’ exposure and vulnerability to mental health problems. These factors may shape developmental experiences, coping mechanisms, and susceptibility to depressive symptoms [23]. Level of education and Socioeconomic Status (SES) are also categorized as structural determinants, representing access to knowledge, resources, and opportunities that may influence psychological well-being. Lower educational attainment and socioeconomic disadvantage are often associated with increased exposure to stressors and limited access to supportive resources [24, 25].

Living condition is conceptualized as an intermediary determinant, reflecting the immediate physical and social environment in which adolescents live. This includes household context and daily living circumstances, which may either contribute to or protect against psychological distress [26]. Family support is also positioned as an intermediary determinant, representing the quality of interpersonal relationships and social support systems. It plays a critical role in shaping emotional resilience and coping capacity [27, 28].

The Stress-Buffering Model of Social Support further strengthens this framework by providing a theoretical explanation for the role of family support. According to this model, family support may act as a protective factor that buffers the negative impact of adverse socioeconomic conditions and stressful life circumstances on depression [29]. In this sense, family support is hypothesized to potentially moderate the relationship between structural determinants (*e.g.*, SES, education) and depressive outcomes.

In addition, the Ecological Systems Theory supports the understanding that adolescent depression arises from interactions across multiple levels of influence. Individual characteristics (*e.g.*, age and gender), microsystem factors (*e.g.*, family support and living conditions), and broader structural conditions (*e.g.*, socioeconomic status and education) are interconnected and jointly shape mental health outcomes [30, 31].

Based on this integrated framework, the study assumes that depression among adolescents is influenced by both structural and intermediary determinants, as well as their interactions. While the present study focuses on examining direct (bivariate) associations, the conceptual model acknowledges the possibility of more complex pathways, including moderating effects of family support on the relationship between socioeconomic and environmental factors and depression.

Numerous studies have investigated the determinants of depression. Nonetheless, research examining the relationship between sociodemographic factors and family support with depression among adolescents in Semarang remains limited, particularly in the student population. Consequently, this study aimed to examine the association between sociodemographic factors and family support with depression among adolescents in Semarang, Indonesia.

## 2 METHOD

### 2.1 Study Design

This study employed quantitative research with a descriptive correlational design and a cross-sectional approach. The study was conducted from March to April 2025 in four high schools in Semarang.

### 2.2 Sample/Participants

The population of the study comprised adolescents in Semarang, Indonesia. The sample of the study consisted of high school students aged 16–18 in the 10th and 11th grades from four selected high schools, whom got permission from their parents or guardians. Student who did not attend school during data collection was excluded from the study. Schools were selected based on having the largest student populations within each administrative area of Semarang (South, East, West, and North Semarang). This approach was used to ensure geographical representation across the city. The sampling method used was proportional stratified random sampling. According to the sample calculation using the Slovin formula, the minimum required sample size was determined to be 421 respondents. The study achieved a 100% response rate, as all eligible participants who were approached agreed to participate and complete the questionnaire.

### 2.3 Instrument

The instruments utilized in this study comprised a Sociodemographic Questionnaire, the Perceived Social Support Family (PSS-Fa), and the Patient Health Questionnaire (PHQ-9). A Sociodemographic Questionnaire was utilized to collect the sociodemographic attributes of the research sample. The tool gathered information about the respondents' age (16, 17, and 18 years old), gender (male and female), living condition (living with parents, living with guardians, and living alone), level of education (10^th^ and 11^th^ grade), and their socioeconomic status (below and above Minimum Wage Rp3,243,969).

The Patient Health Questionnaire (PHQ-9) has been translated into many languages, such as Indonesian. The questionnaire gives each item a score of 0 (not at all), 1 (a few days), 2 (more than half the days), and 3 (almost every day). The total score shows the severity of depression. A score of 0-4 suggests no or minimal depression, 5-9 indicates mild depression, 10-14 signifies moderate depression, 15-19 represents moderately severe depression, and 20-27 denotes severe depression. The Indonesian version of PHQ-9 has demonstrated strong internal consistency (Cronbach’s α = 0.936) and adequate construct validity.

The Perceived Social Support Family (PSS-Fa) questionnaire was used to assess the extent of family support. The PSS-Fa consists of 20 items, employing a Likert scale featuring 15 positive and five negative questions. Positive responses were scored as follows: “yes” receives a score of 3 (indicating family support), “no” receives a score of 2, and “don't know” receives a score of 1. Conversely, for the five negative items, a “no” response is scored as 3, a “yes” as 2, and “don't know” as 1. The total possible score on the questionnaire is 60, with scores categorized into three levels: 20-33 indicating insufficient family support, 34-47 indicating sufficient family support, and 48-60 indicating good family support. The PSS-Fa questionnaire was translated into Indonesian, with a mean validity test result of r=0.361 and a reliability test result using Cronbach's alpha of 0.752. Permission has been obtained from previous researchers for the use of the Perceived Social Support Family (PSS-Fa) questionnaire and the Patient Health Questionnaire (PHQ-9) in the Indonesian context.

### 2.4 Data Collection

Data collection was carried out by distributing questionnaires directly to the respondents at the school. The researcher was accompanied by the class teacher during the distribution of the questionnaires to the students, and if there were any difficulties, the class teacher clarified any questions that the respondents did not understand. Participation of respondents in this study was voluntary, and all respondents were ensured to have obtained written permission from their parents or guardians in the form of informed consent.

### 2.5 Data Analysis

Univariate data analysis was performed to determine the frequency distribution and percentage of each variable. Bivariate analysis was conducted using the chi-square test to assess the correlation among the variables of gender, education level, socioeconomic status, and family support, while Fisher’s exact test was applied to the variable of living conditions. Fisher's exact test was used because the frequency of hope was less than five in the contingency table. A multivariate ordinal logistic regression analysis was performed to assess factors associated with depression status among adolescents.

### 2.6 Research Result

Table **1** presents the sociodemographic data, family support, and depression status of adolescents in Semarang. The study participants were aged between 16 and 18 years. Among these respondents, 260 (61.8%) were 16 years old, 255 (60.6%) were female, and 403 (95.7%) resided with their parents. Additionally, 240 participants (57%) were in the 10th grade, 227 (53.9%) belonged to a higher socioeconomic status, 218 (51.8%) received substantial family support, and 139 (33%) experienced mild depression.

The correlation of sociodemographic factors (gender, education level, living condition, socioeconomic status) and family support with depression level in adolescents is provided in Table **2**.

Table **2** presents the association between sociodemographic factors (gender, education level, living condition, socioeconomic status) and family support with depression levels among adolescents in Semarang (n = 421). The analysis showed that gender was significantly associated with depression level (*p* < 0.001). Female adolescents had a higher proportion of moderate to severe depression compared to males. In contrast, male adolescents were more likely to be categorized as having no or mild depression. A statistically significant association was also found between living condition and depression level (*p* = 0.037). Adolescents living with parents tended to have lower levels of severe depression compared to those living with guardians or non-parents. Notably, adolescents living with guardians showed a relatively higher proportion of severe depression.

In addition, family support demonstrated a strong and significant association with depression level (*p* < 0.001). Adolescents with good family support were more likely to be in the no or mild depression categories, whereas those with sufficient or poor support showed higher proportions of moderate to severe depression. The proportion of severe depression was highest among adolescents with poor family support.

On the other hand, education level (*p* = 0.279) and socioeconomic status (*p* = 0.577) were not significantly associated with depression level. Although some variations in distribution were observed descriptively, these differences were not statistically significant. Overall, these findings indicate that gender, living condition, and family support are significantly associated with depression levels, while education level and socioeconomic status are not significantly related in this study.

A multivariate ordinal logistic regression analysis was performed to assess factors associated with depression status among adolescents. After adjusting for age, gender, socioeconomic status, living condition, and family support, only gender and family support remained significantly associated with depression status. Adolescents with better family support were less likely to experience higher levels of depression, whereas female adolescents showed higher odds of being in more severe depression categories. Other variables were not significantly associated after adjustment. The results of the analysis are provided in Table **3**.

Multivariate ordinal logistic regression analysis revealed that gender and family support were significantly associated with depression status. Female adolescents had higher odds of experiencing more severe depression compared to males (AOR = 1.79, 95% CI: 1.15–2.80, *p* = 0.011). Conversely, adolescents with good family support were less likely to report higher levels of depression (AOR = 0.54, 95% CI: 0.38–0.77, *p* = 0.001). Age, socioeconomic status, and living condition were not significantly associated with depression status after adjustment. The model demonstrated acceptable fit, and the proportional odds assumption was satisfied.

## 3 DISCUSSION

This study seeks to investigate the association between demographic factors and family support in relation to depression among adolescents in Semarang.

### 3.1 The Correlation between Gender and Depression Status among Adolescents

This study found a significant association between gender and depression status among adolescents, with a higher proportion of female participants reporting depressive symptoms compared to males. This finding is consistent with previous studies, which have also reported a higher prevalence of depression among female adolescents [32, 33].

From a biological perspective, puberty-related hormonal fluctuations, particularly involving estrogen and progesterone, have been associated with increased emotional sensitivity and vulnerability to mood disturbances among female adolescents [34–36]. These physiological changes may contribute to a higher susceptibility to depressive symptoms [37]. In addition, psychological factors play an important role in explaining this association. Female adolescents are more likely to engage in ruminative coping, a cognitive style involving repetitive focus on negative thoughts and feelings, which has been strongly linked to depression [38, 39]. They are also more likely to experience concerns related to body image and self-esteem, which may further increase emotional distress [40, 41].

Social and environmental influences are also relevant. Female adolescents tend to report higher exposure to interpersonal stressors, such as peer conflict, relational aggression, and social comparison [42]. These stressors may be intensified by the increasing use of social media, which can amplify feelings of inadequacy and social pressure. Additionally, societal expectations and academic demands may contribute to psychological strain, particularly among female adolescents [43–45].

From a sociocultural perspective, gender norms may influence how emotional distress is experienced and expressed. In many contexts, females may be more likely to acknowledge and report depressive symptoms, whereas males may underreport due to social expectations related to emotional restraint [46-48]. This may partly contribute to the observed differences in prevalence.

### 3.2 The Correlation between Living Condition and Depression Status in Adolescents

This study initially identified a significant association between living condition and depression status among adolescents in the bivariate analysis, suggesting that adolescents’ residential arrangements may be related to their emotional well-being. Most participants in this study resided with their parents, and this finding is consistent with previous studies that have reported an association between living conditions and depressive symptoms in children and adolescents [49–51].

However, after adjusting for potential confounding variables, including age, gender, socioeconomic status, and family support, the association between living condition and depression status was no longer statistically significant in the multivariate analysis. This finding indicates that the relationship observed in the bivariate analysis may be influenced by other underlying factors rather than representing an independent effect of living condition itself.

Previous literature suggests that family-related factors, particularly the quality of parent–adolescent relationships and the level of emotional and instrumental support, play a more critical role in shaping adolescent mental health outcomes than living arrangements alone [52, 53]. For instance, emotional closeness within families and supportive parenting practices have been consistently associated with better psychological well-being [54, 55], whereas less supportive or more demanding family environments have been linked to increased depressive symptoms [56, 57].

Therefore, the present findings suggest that family support may be a more salient determinant of adolescent depression than living conditions [58]. Adolescents who live with their families may still experience depressive symptoms if the quality of support is inadequate. In contrast, those living in alternative arrangements may maintain good mental health when sufficient support systems are present [59, 60].

These findings highlight the importance of considering both structural factors (such as living conditions) and relational factors (such as family support) when examining adolescent mental health. Future studies are recommended to further explore the complex interplay between these variables using longitudinal designs to better understand causal relationships.

### 3.3 The Correlation between Education and Depression in Adolescents

The results of this study indicated that there was no significant association between education level and depression levels among adolescents. Cross-tabulation showed a similar number of moderate depression cases among 10th- and 11th-grade students, suggesting no clear pattern across educational levels within this sample.

This finding differs from a previous study that reported an association between educational attainment and depression, where individuals with higher levels of education tend to report lower levels of depressive symptoms [61, 62].

This study found no significant association between education level and depression among adolescents. This finding suggests that, within the current sample, educational level alone may not be a strong determinant of depressive symptoms [63, 64]. The absence of a statistically significant relationship should be interpreted cautiously, as it does not necessarily indicate that education plays no role in adolescent mental health; rather, it suggests that no clear association was detected in this study.

Several explanations may account for this finding. First, the relatively homogeneous educational context of the participants may have limited variability in academic exposure and experiences, thereby reducing the ability to detect differences in depression across education levels [65]. Second, adolescents’ mental health may be more strongly influenced by proximal psychosocial factors, such as family support, peer relationships, and living conditions, which could outweigh the role of education level as a distal factor [66–68]. Third, the measurement of education in this study may not have fully captured relevant aspects of the educational experience. Education level as a categorical variable may overlook important dimensions such as academic pressure, school environment, learning difficulties, and perceived academic performance, all of which have been shown to be more directly related to depressive symptoms. Therefore, the lack of association may reflect measurement limitations rather than the absence of a true relationship.

Additionally, it is possible that the relationship between education and depression is indirect or context-dependent, potentially mediated or moderated by other variables such as socioeconomic status or social support. The current cross-sectional design and analytical approach may not have been sufficient to capture these more complex pathways. Overall, these findings highlight that education level alone may not be an adequate indicator of adolescent mental health risk.

### 3.4 The Correlation between Socioeconomic Status and Depression Level in Adolescents

In this study, most adolescents were from families with relatively high socioeconomic status. The present study found no statistically significant association between Socioeconomic Status (SES) and depression levels among adolescents. This finding indicates that socioeconomic differences may not directly influence depressive symptoms within the study population.

This result contrasts with a growing body of evidence from Southeast Asian countries, where socioeconomic disadvantage is often identified as a significant determinant of depression. A recent systematic review focusing on low- and middle-income countries in Southeast Asia reported that lower SES is consistently associated with a higher risk of depression, highlighting financial hardship and limited access to resources as key contributing factors [69]. These findings suggest that, in many Southeast Asian contexts, SES plays an important role in shaping mental health outcomes.

However, the current finding aligns with emerging evidence indicating that the relationship between SES and adolescent depression is not always direct. Some studies suggest that socioeconomic factors may influence mental health indirectly through mediating variables such as family functioning, parenting practices, and peer relationships. For example, research in Asian populations has shown that SES affects adolescent mental health through mechanisms such as parent–child interaction and social relationships rather than through a direct pathway [70]. This indirect effect may explain why no statistically significant association was observed in the present study.

In addition, inconsistencies across Southeast Asian studies may reflect contextual and cultural differences. In collectivist societies, including Indonesia and many Southeast Asian countries, strong family cohesion and community-based social support systems may buffer the negative impact of low socioeconomic status. These protective factors can reduce adolescents’ vulnerability to depression, even in economically disadvantaged conditions. As a result, the direct effect of SES may become less apparent when social support is strong.

Another important consideration is the distinction between objective and subjective socioeconomic status. Evidence suggests that subjective perceptions of economic position may have a stronger association with depression than objective indicators such as income or parental education [70]. If the present study relied primarily on objective SES measures, it is possible that the psychosocial dimensions of perceived inequality were not fully captured, contributing to the non-significant findings.

Furthermore, adolescent depression is a multifactorial phenomenon influenced by proximal factors such as gender, academic pressure, peer relationships, and individual coping strategies. In Southeast Asian contexts, these factors may play a more immediate role than distal determinants like SES. For instance, studies in Indonesia have identified behavioral and health-related factors, rather than socioeconomic variables, as predictors of depression [12]. This supports the notion that SES alone may not be sufficient to explain variations in adolescent depression.

### 3.5 The Correlation between Family Support and Depression Level in Adolescents

This study identified a statistically significant association between family support and depression levels among adolescents. Adolescents who reported higher levels of family support tended to report lower levels of depressive symptoms. This finding indicates that family support is closely related to adolescents’ mental health status.

The observed association is consistent with recent empirical studies. For example, a previous study highlights the importance of family support and the adoption of adaptive coping mechanisms in promoting adolescent mental health, including depression [71]. Similarly, another study reported that family support was significantly correlated with reduced depressive symptoms in adolescents, although the authors emphasized that the relationship may be influenced by other psychosocial factors [72]. In addition, adolescents with stronger perceived family support reported better psychological well-being and fewer depressive symptoms [73].

Importantly, this relationship should be interpreted as an association rather than a causal effect. The cross-sectional design of the present study limits the ability to determine whether low family support contributes to depression or whether adolescents experiencing depressive symptoms perceive or report lower levels of family support. This possibility of bidirectionality has been noted in previous research. Adolescents with depressive symptoms may interpret family interactions more negatively, thereby reporting lower levels of perceived support [74].

Furthermore, the association between family support and depression may be shaped by other contextual and psychosocial variables. Studies have shown that factors such as peer support, academic stress, and coping strategies may interact with family support in influencing depressive symptoms. The relationship between family support and depression was partially mediated by loneliness and moderated by peer relationships, indicating a complex interplay of interpersonal influences [75].

From a conceptual perspective, these findings are consistent with broader frameworks such as the Social Determinants of Health and Ecological Systems Theory, which emphasize that adolescent mental health is influenced by multiple interconnected systems. Within this framework, family support represents an important interpersonal factor associated with mental health outcomes, but not a sole determinant.

Additionally, recent literature emphasizes the importance of perceived family support. Subjective perceptions such as feeling understood, valued, and emotionally supported are more strongly associated with depressive symptoms than objective indicators of family characteristics. Perceived emotional support from family members had a stronger relationship with depressive symptoms than structural family variables [75].

## 4 LIMITATION AND IMPLICATION

This study has several limitations that should be considered when interpreting the findings. The use of a cross-sectional design limits the ability to establish causal relationships between the identified factors and depression. The data were collected using self-reported questionnaires, which may be subject to recall bias and social desirability bias. The study employed a school-based sampling approach, which may limit the generalizability of the findings to adolescents who are not enrolled in school or who have dropped out. Depression was assessed using the PHQ-9 screening instrument rather than a formal clinical diagnostic evaluation, which may not fully capture clinically diagnosed depressive disorders. This study has incorporated a multivariate ordinal logistic regression analysis to adjust for key variables; there remains the possibility of residual confounding from unmeasured factors that were not included in the model. The majority of participants were living with their parents (n=403), while only a small proportion lived with guardians (n=16) or alone (n=2). The extremely small sample sizes in the latter categories may limit the statistical reliability and stability of the estimates, and therefore the observed associations involving these groups should be interpreted with caution. This imbalance may also reduce the generalizability of the findings regarding adolescents who do not live with their parents. Another limitation of this study is that data were collected only from students who were present at school during the survey administration. Students who were absent were not included in the sample. Considering that previous research has shown an association between depression and school absenteeism, it is possible that adolescents experiencing more severe depressive symptoms were underrepresented in this study. As a result, the prevalence of depression reported in this study may be underestimated. The data from this study can be used by nurses or other health workers as a basis for planning mental health programs for adolescents.

## CONCLUSION AND RECOMMENDATION

The findings indicate that most participants were aged between 16 and 18 years, predominantly female, living with their parents, enrolled in the 10th grade, had a socioeconomic status above the minimum wage, and reported high levels of family support. A considerable proportion of adolescents experienced moderate to severe depression. The study identified significant associations between gender, living conditions, and family support and depression among adolescents in Semarang. In contrast, no significant associations were found between level of education, socioeconomic status, and depression. Given the cross-sectional design of this study, the findings should be interpreted as associative rather than causal. Future research is recommended to explore additional contributing factors and to consider longitudinal or qualitative approaches to better understand adolescent mental health. Besides, future studies are encouraged to include a more balanced representation of living conditions to allow for more robust and reliable comparisons, and consider alternative data collection strategies to include students who are absent, in order to obtain a more comprehensive assessment of adolescent mental health.

## Figures and Tables

**Table 1:** Frequency distribution of sociodemographic factors, family support, and depression status among adolescents in semarang (n=421).

**Sociodemographic Variable, Family Support, and Depression Status**	**Frequency (n)**	**Percentage (%)**	**Mean**	**Std. Deviation**
**Age**				
16 Years Old	260	61.8	16.42	0.557
17 Years Old	147	34.9		
18 Years Old	14	3.3		
**Sex**				
Male	166	39.4	-	-
Female	255	60.6	-	-
**Living Condition**				
Living with parents	403	95.7	-	-
Living with guardians/non-parents	16	3.8	-	-
Living alone	2	0.5	-	-
**Education Level**				
10th Grade	240	57	-	-
11th Grade	181	43	-	-
**Socioeconomic Status**	-	-	-	-
Below Minimum Wage (<Rp3,243,969)	194	46.1	-	-
Above Minimum Wage (>Rp3.243.969)	227	53.9	-	-
**Family Support**				
Good	218	51.8	-	-
Sufficient	181	43.0	-	-
Poor	22	5.2	-	-
**Depression Level**				
No depression	119	28.2	8.43	5.53
Mild depression	139	33.0	-	-
Moderate depression	98	23.3	-	-
Moderately severe depression	50	11.9	-	-
Severe depression	15	3.6	-	-

**Table 2:** Correlation of sociodemographic factors (gender, education level, living condition, socioeconomic status) and family support with depression level in adolescents in semarang (n=421).

	**Depression Level *f* (%)**					
**The Variable of Sociodemographic Factors and Family Support**	No Depression	Mild Depression	Moderate Depression	Moderately Severe Depression	Severe Depression	***P*-value**
**Gender**						
Male	67 (40.4%)	52 (31.3%)	36 (21.7%)	8 (4.8%)	3 (1.8%)	0.000*
Female	52 (20.4%)	87 (34.1%)	62 (24.3%)	42 (16.5%)	12 (4.7%)	-
**Living Condition**						
Living with Parents	116 (28.8%)	132 (32.8%)	95 (23.6%)	48 (11.9%)	12 (3.0%)	0.037*
Living with Guardians/Non-Parents	3 (18.8%)	7 (43.8%)	2 (12.5%)	1 (6.2%)	3 (18.8%)	-
Living Alone	0 (0%)	0 (0%)	1 (50%)	1 (50%)	0 (0%)	-
**Education Level**						
10th Grade	65 (27.1%)	88 (36.7%)	49 (20.4%)	28 (11.7%)	10 (4.2%)	0.279
11th Grade	54 (29.8%)	51 (28.2%)	49 (27.1%)	22 (12.2%)	5 (2.8%)	-
**Socioeconomic Status**						
Below Minimum Wage (<Rp3,243,969)	53 (27.3%)	60 (30.9%)	45 (23.2%)	26 (14.4%)	8 (4.1%)	0.577
Above Minimum Wage (>Rp3,243,969)	66 (29.1%)	79 (34.8%)	53 (23.3%)	22 (9.7%)	7 (3.1%)	-
**Family Support**						
Good Support	82 (37.6%)	76 (34.9%)	38 (17.4%)	20 (9.2%)	2 (0.9%)	0.000*
Sufficient Support	31 (17.1%)	59 (32.5%)	55 (30.4%)	26 (14.4%)	10 (5.5%)	
Poor Support	6 (27.3%)	4 (18.2%)	5 (22.7%)	4 (18.2%)	3 (13.6%)	

**Note:** The Chi-Square Test (Gender, education level, socioeconomic status, family support) *sig.(*p*<0.05) Fisher Exact Test (living condition), *sig.(*p*<0.05).

**Table 3:** Factors associated with depression status among adolescents after adjusting for age, gender, socioeconomic status, living condition, and family support in semarang (n=421).

Variables	β Coefficient	Adjusted Odds Ratio (AOR)	95% Confidence Interval	*P*-value
Age	0.07	1.07	0.95 – 1.21	0.241
Gender	0.58	1.79	1.15 – 2.80	0.011*
SES	0.29	1.34	0.91 – 1.97	0.134
Living condition	0.18	1.20	0.82 – 1.75	0.352
Family support	-0.62	0.54	0.38 – 0.77	0.001*

**Note:** A multivariate ordinal logistic regression analysis *sig.(*p*<0.05).

## Data Availability

The data supporting the findings of this article are available in the Zenodo Repository at https://zenodo.org/ records/21347199, with reference number (DOI): https://doi.org/10.5281/zenodo.21347199.

## LIST OF ABBREVIATIONS

PSS-Fa: Perceived Social Support Family
PHQ-9: Patient Health Questionnaire-9

## STANDARDS OF REPORTING

STROBE guidelines were followed.

## References

[1] Backes E.P., Bonnie R.J. (2019). Adolescent development.. The Promise of Adolescence: Realizing Opportunity for All Youth.

[2] Mastorci F., Lazzeri M.F.L., Vassalle C., Pingitore A. (2024). The transition from childhood to adolescence: Between health and vulnerability.. Children.

[3] Hendrickx G., De Roeck V., Maras A., Dieleman G., Gerritsen S., Purper-Ouakil D., Russet F., Schepker R., Signorini G., Singh S.P., Street C., Tuomainen H., Tremmery S. (2020). Challenges during the transition from child and adolescent mental health services to adult mental health services.. BJPsych Bull..

[4] Pham M.D., Wulan N.R., Sawyer S.M., Agius P.A., Fisher J., Tran T., Medise B.E., Devaera Y., Riyanti A., Ansariadi A., Cini K., Kennedy E., Wiweko B., Luchters S., Kaligis F., Wiguna T., Azzopardi P.S. (2024). Mental health problems among indonesian adolescents: Findings of a cross-sectional study utilizing validated scales and innovative sampling methods.. J. Adolesc. Health.

[5] Thapar A., Collishaw S., Pine D.S., Thapar A.K. (2012). Depression in adolescence.. Lancet.

[6] Az-Zahra F., Setiawati Y., Umijati S. (2021). The correlation between depression and self-harm behavior in teenagers: A systematic narrative review.. Int. J. Creat. Res. Thoughts..

[7] Tjandrarini D.H., Ashar H., Nari J.P., Malakauseya M.L.V., Senewe F.P., Musoddaq M.A., Nazarina N., Mulyantoro D.K., Wardhani Y.F., Izza N., Titaley C.R. (2025). Disabilities and depression in young adolescents living in underdeveloped areas of Indonesia: Results from the 2018 Indonesia basic health survey.. BMJ Open.

[8] Idris H., Tuzzahra F. (2023). Factors associated with depressive symptoms among adolescents in Indonesia: A cross-sectional study of results from the Indonesia Family Life Survey.. Malays. Fam. Physician.

[9] Chi X., Liu X., Huang Q., Huang L., Zhang P., Chen X. (2020). Depressive symptoms among junior high school students in Southern China: Prevalence, changes, and psychosocial correlates.. J. Affect. Disord..

[10] Lewandowska A., Bliźniewska-Kowalska K., Gałecki P., Kubiak R. (2022). Experiencing violence among children and adolescents with depression in the aspect of Polish law.. J. Clin. Med..

[11] Remes O., Mendes J.F., Templeton P. (2021). Biological, psychological, and social determinants of depression: A review of recent literature.. Brain Sci..

[12] Purborini N., Lee M.B., Devi H.M., Chang H.J. (2021). Associated factors of depression among young adults in Indonesia: A population-based longitudinal study.. J. Formos. Med. Assoc..

[13] D’Onofrio B., Emery R. (2019). Parental divorce or separation and children’s mental health.. World Psychiatry.

[14] Juliansen A., Heriyanto R.S., Muljono M.P., Budiputri C.L., Sagala Y.D.S., Octavius G.S. (2024). Mental health issues and quality of life amongst school-based adolescents in Indonesia.. J. Med. Surg. Public Health.

[15] Reiss F., Meyrose A.K., Otto C., Lampert T., Klasen F., Ravens-Sieberer U. (2019). Socioeconomic status, stressful life situations and mental health problems in children and adolescents: Results of the German BELLA cohort-study.. PLoS One.

[16] Muttaqin Z., Muryati M., Purnama D., Rukman R. (2022). Family support for healing mental disorder patients with social isolation in the work area of Pasirkaliki public health center, Bandung city.. Maj. Kesehat. Indones..

[17] Fitzpatrick M.M., Anderson A.M., Browning C., Ford J.L. (2024). Relationship between family and friend support and psychological distress in adolescents.. J. Pediatr. Health Care.

[18] Spector A.Y. (2019). Social determinants of health in the human services: A conceptual model and road map for action.. J. Prog. Hum. Serv..

[19] Taylor M., Hilario C.T., Ben-David S., Dimitropoulos G. (2024). A social determinants perspective on adolescent mental health during the COVID-19 pandemic.. COVID.

[20] Wimmer J., Coyle-Eastwick S., Fox J.K., Grapin S. (2025). Stress, social support, and internalizing problems: Domains of stress and support.. Psychol. Sch..

[21] Foo Y.Y., Goy R., Nestel D., Reedy G., McKenna L., Gough S. (2023). Clinical Education for the Health Professions: Theory and Practice..

[22] Hassan I., Chisty A., Bui T. (2024). Structural and social determinants of health.. Leading an Academic Medical Practice.

[23] Sánchez-Hernández Ó., Méndez F.X., Marín-Martínez F. (2023). Gender, age and depressive symptoms in adolescence.. Psicol. Conduct..

[24] Lee K.S., Yang Y. (2022). Educational attainment and emotional well-being in adolescence and adulthood.. SSM Ment. Health.

[25] Liu K., Liu J. (2023). Effect of subjective and objective socioeconomic status on physical health, mental health, and well-being.. Soc. Behav. Personal..

[26] Morillo-Sarto H., Torres-Vallejos J., Usán P., Barrada J.R., Juarros-Basterretxea J. (2025). The impact of social media disorder, family functioning, and community social disorder on adolescents’ psychological distress: The mediating role of intolerance to uncertainty.. Children.

[27] Yan Y., Duan X., Tan Y., Wu T., Yang B.X., Luo D., Liu L. (2025). The relationship between family functioning and depressive symptoms: Mediating effects of psychological resilience and parent-child interactions.. J. Affect. Disord..

[28] Ao L., Cheng X., An D., An Y., Yuan G. (2025). Relationship between perceived family resilience, emotional flexibility, and anxiety symptoms: A parent–adolescent dyadic perspective.. J. Youth Adolesc..

[29] Pössel P., Burton S.M., Cauley B., Sawyer M.G., Spence S.H., Sheffield J. (2018). Associations between social support from family, friends, and teachers and depressive symptoms in adolescents.. J. Youth Adolesc..

[30] Lin Y., Jia G., Zhao Z., Li M., Cao G. (2024). The association between family adaptability and adolescent depression: The chain mediating role of social support and self-efficacy.. Front. Psychol..

[31] Zeng Z.Y., Ye W.Y., He Y.Z., Gu W.H., Li S.N., Nie Y.G. (2025). Longitudinal association between parent–child relationship and depression among Chinese adolescents: The role of psychological resilience and school climate.. Child Adolesc. Psychiatry Ment. Health.

[32] Toro G.V.R., Arias P., de la Torre-Luque A., Singer J.B., Lagunas N. (2025). Depression, anxiety, and suicide among adolescents: Sex differences and future perspectives.. J. Clin. Med..

[33] Liu D., Lei G., Li D., Deng H., Zhang X.Y., Dang Y. (2024). Depression, rumination, and suicide attempts in adolescents with mood disorders.. J. Clin. Psychiatry.

[34] Neitzke E.V., dos Santos F.G., Zanini B.M., Cavalcante M.B., Mason J.B., Masternak M.M., de Souza I.C.C., Schneider A. (2025). The influence of ovarian activity and menopause on mental health: Evidence from animal models and women.. Physiol. Behav..

[35] Andersen E., Klusmann H., Eisenlohr-Moul T., Baresich K., Girdler S. (2024). Life stress influences the relationship between sex hormone fluctuation and affective symptoms in peripubertal female adolescents.. Dev. Psychopathol..

[36] Andersen E., Prim J., Campbell A., Schiller C., Baresich K., Girdler S. (2024). Biobehavioral mechanisms underlying testosterone and mood relationships in peripubertal female adolescents.. Dev. Psychopathol..

[37] Graham B.M., Denson T.F., Barnett J., Calderwood C., Grisham J.R. (2018). Sex hormones are associated with rumination and interact with emotion regulation strategy choice to predict negative affect in women following a sad mood induction.. Front. Psychol..

[38] Hevron H., Weinbach N. (2024). Self-compassion and cognitive reappraisal restore female adolescents’ body satisfaction and appreciation after appearance-related rumination.. Body Image.

[39] Miloseva L., Vukosavljevic-Gvozden T., Milosev V., Davis T. (2018). Can we predict and prevent subclinical depression in adolescents?. J. Nerv. Ment. Dis..

[40] Maezono J., Hamada S., Sillanmäki L., Kaneko H., Ogura M., Lempinen L., Sourander A. (2019). Cross‐cultural, population‐based study on adolescent body image and eating distress in Japan and Finland.. Scand. J. Psychol..

[41] Kim S.Y., Eo Y.S. (2026). Self-Esteem as a mediator between body-esteem and depression among Korean adolescents: Differences by weight status.. Healthcare.

[42] Díaz-Moreno A., Bonilla I., Chamarro A. (2023). Negative social comparison: The influence of anxiety, emotion regulation and problematic social media use.. Ansiedad Estres.

[43] Jin L.T., Choi Y.J. (2026). Are your defense mechanisms harming you? The role of psychological defenses in social media comparison and adolescent self-esteem.. Comput. Human Behav..

[44] Dongre S.A., Bhandarkar T.D., Tidke D.M. (2024). Effects of social media on mental health on higher secondary school students.. MSW Manag..

[45] Feng N., Yang Y., Xie Z., Qiu T., Cui L. (2024). Longitudinal study examining academic expectation stress and distrust for Chinese adolescents: The roles of internalizing problems and hope.. Soc. Dev..

[46] Thayer J.F., Rossy L.A., Ruiz-Padial E., Johnsen B.H. (2003). Gender differences in the relationship between emotional regulation and depressive symptoms.. Cognit. Ther. Res..

[47] Koenig L.R., Blum R.W., Shervington D., Green J., Li M., Tabana H., Moreau C. (2021). Unequal gender norms are related to symptoms of depression among young adolescents: A cross-sectional, cross-cultural study.. J. Adolesc. Health.

[48] Smith D.S., Schacter H.L., Enders C., Juvonen J. (2018). Gender norm salience across middle schools: Contextual variations in associations between gender typicality and socioemotional distress.. J. Youth Adolesc..

[49] Zhou C., Dai L., He Y., Zhu J. (2023). Social support and parent-child relationship among adolescents and their influence on emotions.. Shanghai J Prev Med..

[50] Manczak E.M., Ordaz S.J., Singh M.K., Goyer M.S., Gotlib I.H. (2019). Time spent with parents predicts change in depressive symptoms in adolescents with major depressive disorder.. J. Abnorm. Child Psychol..

[51] Janssen L.H.C., Verkuil B., van Houtum L.A.E.M., Wever M.C.M., Wentholt W.G.M., Elzinga B.M. (2024). A closer look into the affect dynamics of adolescents with depression and the interactions with their parents: An ecological momentary assessment study.. Eur. Child Adolesc. Psychiatry.

[52] Morgan D.D., Higgins C.D., Rogers C.R. (2025). The role of parent’s mental health in shaping perceptions of adolescent mental health experiences after COVID ‐19.. Fam. Relat..

[53] Xu T., Ren J. (2025). Parent–child relationships, parental control, and adolescent mental health: An empirical study based on CEPS 2013–2014 survey data.. Behav. Sci..

[54] Chen J., Zhou X. (2021). Within-family patterns of intergenerational emotional closeness and psychological well-being of older parents in China.. Aging Ment. Health.

[55] Chen L., Fosco G.M., Tornello S.L. (2024). Who benefits from autonomy-supportive parenting? Considering individual difference in adolescent emotional reactivity.. J. Child Fam. Stud..

[56] Liao S., Wang D. (2025). Patterns of family–school environment influence depressive symptoms among adolescents in China: The mediating role of resilience.. Front. Psychiatry.

[57] Ren M., Song J., Zhou C., Hou J., Huang H., Li L. (2024). Effects of family environment on depressive symptoms in postgraduate students: Longitudinal moderating effect of family support and mediating effect of psychological resilience.. Depress. Anxiety.

[58] Tian G., Wang J., Zhu J., Hu H., Hao Y. (2025). Associations of family support and loneliness with underlying depression in Chinese children and adolescents.. BMC Public Health.

[59] Ding J., Wan Q., Gan M., Yin X., Wu H., Ma Y. (2023). Correlation between family environment and depressive symptoms among adolescents.. Chin J Sch Health..

[60] Sun F., Wang Y., Li W., Li M., Hui T., Shi W. (2025). Exploring the influence of living arrangements on mental health among adolescents during the COVID-19 pandemic: A cross-sectional study in China.. BMC Public Health.

[61] Chlapecka A., Kagstrom A., Cermakova P. (2020). Educational attainment inequalities in depressive symptoms in more than 100,000 individuals in Europe.. Eur. Psychiatry.

[62] Tassone V.K., Duffy S.F., Dunnett S., Boparai J.K., Zuluaga Cuartas V., Jung H., Wu M., Goel N., Lou W., Bhat V. (2024). Decreased odds of depressive symptoms and suicidal ideation with higher education, depending on sex and employment status.. PLoS One..

[63] Rajan V., Marimuthu Y., Menon V., Kumar Saya G., Raj R. (2024). Effect of spiritual intelligence and employment status on the association between education and depressive symptoms among adults in rural Puducherry, India: A mediation analysis.. Int. J. Soc. Psychiatry.

[64] Zhao R., Wang J., Lou J., Liu M., Deng J., Huang D., Fang H. (2024). The effect of education level on depressive symptoms in Chinese older adults–parallel mediating effects of economic security level and subjective memory ability.. BMC Geriatr..

[65] Li Y., Zhao D. (2021). Education, neighbourhood context and depression of elderly Chinese.. Urban Stud..

[66] Wang M.T., Toro J.D., Scanlon C.L., Schall J.D., Zhang A.L., Belmont A.M., Voltin S.E., Plevniak K.A. (2021). The roles of stress, coping, and parental support in adolescent psychological well-being in the context of COVID-19: A daily-diary study.. J. Affect. Disord..

[67] Aboagye R.G., Ahinkorah B.O., Seidu A.A., Okyere J., Frimpong J.B., Kumar M. (2022). In-school adolescents' loneliness, social support, and suicidal ideation in sub-Saharan Africa: Leveraging global school health data to advance mental health focus in the region.. PLoS One..

[68] Wiedermann C.J., Barbieri V., Reismann H., Piccoliori G., Engl A., Hager von Strobele-Prainsack D. (2026). Perceived financial strain and adolescent mental health: Evidence from a population-based study in South Tyrol, Italy.. Children.

[69] Barrass L., Joshi E., Dawe J., Rubbo B., Redaniel M.T., Riglin L., Lee N.R., Howe L.D., Knipe D. (2024). The association between socioeconomic position and depression or suicidal ideation in low- and middle-income countries in Southeast Asia: A systematic review and meta-analysis.. BMC Public Health.

[70] Yang D., Hu S., Li M. (2022). The influence of family socioeconomic status on adolescents’ mental health in China.. Int. J. Environ. Res. Public Health.

[71] Jiang M., Yang H. (2026). Family functioning and adolescent depression: Parallel and serial mediation roles of academic stress and emotion regulation.. Behav. Sci..

[72] Wang M.T., Degol J.L., Henry D.A. (2019). An integrative development-in-sociocultural-context model for children’s engagement in learning.. Am. Psychol..

[73] (2023). Family support and adolescent mental health: A cross-sectional study in Asia.. https://www-scopus-com.proxy.undip.ac.id/pages/ai?temporary-convo=false&conversationId=26fa3608-09a3-42de-9300-6ca85bf47ccd.

[74] Boele S., Nelemans S.A., Denissen J.J.A., Prinzie P., Bülow A., Keijsers L. (2023). Testing transactional processes between parental support and adolescent depressive symptoms: From a daily to a biennial timescale.. Dev. Psychopathol..

[75] Liu T., Zhou Z., Zhang S., Zhang F., Yan C., Chen Z. (2026). Latent profiles of perceived social support among adolescents and their relationship with depressive symptoms.. Front. Psychol..

